# Natural killer cell dysfunction in hepatic alveolar echinococcosis: an under-recognized cytotoxic node in parasite immune escape

**DOI:** 10.3389/fimmu.2026.1976611

**Published:** 2026-10-19

**Authors:** Shilong Wang, Kerong Lu, Chao Jiang, Yongqiang Zou, Dalin Shi, Mingquan Pang, Cipeng Fan, Zhixin Wang, Manjun Deng, Chengwei Tie, Haining Fan

**Affiliations:** 1 Qinghai University, Xining, China; 2 Qinghai University Affiliated Hospital, Xining, China; 3 Qinghai Institute for Endemic Disease Prevention and Control, Xining, China; 4 Department of Hepatobiliary and Pancreatic Surgery, Qinghai University Affiliated Hospital, Xining, Qinghai, China

**Keywords:** alveolar echinococcosis, *Echinococcus multilocularis*, fibrosis, hepatic immune microenvironment, immune escape, natural killer cells, NKG2A, TIGIT

## Abstract

Alveolar echinococcosis (AE), caused by the larval stage of *Echinococcus multilocularis*, is a chronic and infiltrative hepatic helminth infection characterized by tumor-like growth, progressive tissue destruction and long-term host-parasite coexistence. Parasite persistence in the liver is sustained not only by local invasion but also by a progressively remodeled immune microenvironment. Most immunological studies of AE have focused on macrophages, neutrophils, regulatory T cells, dendritic cells, T-cell exhaustion and fibrosis. By contrast, natural killer (NK) cells have remained a relatively under-integrated component of AE immunology, despite the liver being enriched in NK and innate lymphoid populations. Accumulating evidence indicates that NK activity is reduced in AE patients, that *E. multilocularis* vesicular fluid can suppress NK-cell activation and proliferation, and that inhibitory receptors including NKG2A and TIGIT are associated with impaired NK-derived IFN-γ, TNF-α, granzyme B and perforin responses in patients and experimental models. Recent work on checkpoint signaling, suppressive immune niches and fibrotic spatial organization further suggests that NK-cell dysfunction should not be interpreted as an isolated NK-intrinsic event. Instead, it may represent an under-recognized cytotoxic node within a parasite-induced hepatic immune escape network shaped by parasite-derived products, myeloid remodeling, Treg/DC crosstalk, inhibitory receptor-ligand interactions and fibrotic immune segregation. In this review, we synthesize the evidence for NK-cell dysfunction in AE, compare the strength of support for NKG2A, TIGIT, inhibitory KIRs and TIM-3/LAG-3-related pathways, and propose an NK-oriented framework for integrating cytotoxic immune dysfunction with parasite persistence and fibrosis-associated lesion remodeling. We also highlight unresolved questions regarding direct versus indirect antiparasitic NK functions, liver-resident NK cells, NK-ILC1-NKT boundaries, receptor-ligand sources and checkpoint-targeted immunomodulation in AE.

## 1 Introduction

Alveolar echinococcosis is among the most severe human cestode infections and is caused by the metacestode stage of *Echinococcus multilocularis*. In humans, the disease primarily affects the liver, where parasite tissue grows slowly but invasively, producing infiltrative lesions that can resemble malignant tumors in local expansion, vascular involvement and destructive tissue remodeling (1, 2). Curative treatment generally requires complete surgical resection combined with prolonged benzimidazole therapy, but many patients are diagnosed at advanced stages, when radical surgery is difficult or impossible and long-term medical treatment becomes necessary (1, 3). These clinical features make AE not only a parasitic disease but also a model of chronic host-parasite coexistence within a highly immunologically active organ.

The liver maintains a distinctive balance between immune surveillance and tolerance. It receives portal venous blood rich in microbial products and dietary antigens, contains abundant innate and innate-like lymphocytes, and must limit excessive inflammatory damage while preserving antimicrobial defense. This tolerogenic bias can be protective, but it may also be exploited by persistent pathogens and parasites. In AE, parasite growth is surrounded by granulomatous inflammation, necrosis, fibrosis, vascular and stromal remodeling, and immune-cell infiltration (2, 4). Early responses can include macrophage activation, neutrophil recruitment, Th1/Th17-type inflammation and IFN-γ-associated host defense. However, progressive disease is frequently associated with immunoregulatory mechanisms, including regulatory T-cell expansion, IL-10 and TGF-β production, checkpoint receptor expression, myeloid suppressor activity and fibrotic lesion encapsulation (4, 5).

For this reason, the immunology of AE has often been discussed through macrophage polarization, neutrophil responses, T-cell exhaustion, regulatory T cells, dendritic-cell dysfunction and fibrosis. These areas are central to the field and have generated important mechanistic insights. Yet they do not fully explain how cytotoxic innate immunity is weakened within the periparasitic liver niche. NK cells are particularly relevant to this question because they are enriched in the liver and can mediate anti-infective responses through IFN-γ, TNF-α, perforin, granzyme B and death-receptor pathways. In AE, however, NK cells should not be assumed to directly kill metacestode tissue. A more defensible model is that NK cells contribute to antiparasitic immunity by sustaining inflammatory cytokine tone, shaping macrophage activation, modulating T-cell responses and influencing lesion remodeling. Despite this biological relevance, NK cells in AE have often appeared as scattered observations rather than as a coherent theme.

Recent evidence increasingly supports closer integration of NK-cell biology into current models of AE immunology. Early clinical work reported reduced NK activity in peripheral blood mononuclear cells from AE patients and described immune-cell organization in periparasitic granulomas (6). Later studies showed that *E. multilocularis* vesicular fluid can suppress NK-cell activation and proliferation (7), induce checkpoint-related proteins *in vitro* (8), and that hepatic NK cells in infected mice exhibit reduced frequency and IFN-γ production together with increased NKG2A expression (9). More recent work has linked TIGIT to NK-cell dysfunction and immune escape in both patients and mouse models (10), identified inhibitory KIR-related changes (11), and provided human tissue and blockade evidence for NKG2A-associated NK dysfunction (12, 13). These studies suggest that NK cells are not incidental bystanders in AE but an under-recognized cytotoxic node in parasite immune regulation. However, current evidence remains uneven and is strongest for NKG2A and TIGIT, whereas many proposed microenvironmental crosstalk mechanisms remain inferential.

Here, we review evidence for NK-cell dysfunction in hepatic AE and discuss how parasite-derived products, inhibitory receptor signaling, myeloid remodeling, Treg/DC crosstalk, CD8 T-cell exhaustion and fibrosis may converge to create a hepatic niche permissive for parasite persistence. Accordingly, NK-cell dysfunction is considered here as an under-recognized cytotoxic node within the established myeloid-, Treg-, T-cell-, and fibrosis-associated immune escape network of hepatic AE. In this review, immune escape is used to describe parasite persistence under incomplete cytotoxic control and regulatory tissue remodeling, rather than the absence of host immune containment. Direct NK evidence is therefore distinguished from tissue-level evidence that supports, but does not yet establish, NK-suppressive crosstalk.

## 2 Literature search strategy for this narrative review

A narrative literature search was conducted in PubMed and the Web of Science Core Collection, focusing on publications from January 1, 2016 through May 27, 2026. Disease terms were combined with terms for NK cells and innate lymphoid cells, inhibitory receptors and their ligands, myeloid and regulatory cell populations, fibrosis and extracellular matrix remodeling, single-cell or spatial immune profiling, and immune dysfunction or suppression. PubMed searches used Title/Abstract fields, whereas Web of Science searches used the Topic field. The complete database-specific search strings are provided below. The publication-date range was specified in the PubMed query and checked during screening for both databases, using the earliest verifiable online or print publication date. Reports first published after May 27, 2026 were excluded.

The database exports comprised 206 PubMed records and 225 Web of Science records. Matching by DOI and normalized article title identified 177 cross-database duplicates, leaving 254 unique records, all of which were screened. Following relevance and publication-date assessment, 165 publications met the broad eligibility criteria and formed the database-derived literature pool: 129 original reports addressing AE-associated immune or tissue-microenvironment findings and 36 contextual or background publications. Among these 165 publications, 133 were identified in both databases, 17 were identified only in PubMed, and 15 were identified only in Web of Science. The remaining 89 records did not meet the criteria for retention as separate publications. These counts describe the database-derived literature pool rather than the number of references cited in the review. References cited in the narrative synthesis were drawn from the database-derived literature pool and from foundational publications identified outside the database exports, with selection guided by their direct thematic and mechanistic relevance to the topics discussed.

The literature synthesis focused on publications relevant to NK-cell dysfunction, inhibitory receptor signaling, and the hepatic immune microenvironment in AE. Studies addressing T-cell exhaustion, regulatory T cells, myeloid cells, dendritic cells, fibrosis, and spatial immune organization were considered when they provided mechanistic or tissue-level context relevant to NK-cell dysfunction or broader cytotoxic immune regulation. Human, animal and *in vitro* studies were considered, including English- and Chinese-language publications. Clinical cohorts, reviews and selected host-parasite studies were retained for context. Records were assessed for thematic relevance, publication date and report type. Records outside the thematic scope, single-case reports without independent immune or tissue-mechanistic findings, retracted publications, and records lacking sufficient content for assessment were not retained. Correction notices and a related conference report were linked to their corresponding articles and were not counted as additional retained publications.

Earlier foundational work was considered separately when it provided key clinical or conceptual evidence, particularly the early report of reduced NK activity in AE patients (6), classic reviews on *E. multilocularis* host-parasite interplay (2, 4), and selected foundational literature on liver NK cells, innate lymphoid cells, NK dysfunction and checkpoint receptor biology. Publications identified outside the database exports were not included in the database-derived screening counts.

### 2.1 PubMed search string

(“alveolar echinococcosis”[Title/Abstract] OR “hepatic alveolar echinococcosis”[Title/Abstract] OR “Echinococcus multilocularis”[Title/Abstract] OR “E. multilocularis”[Title/Abstract]) AND (“natural killer”[Title/Abstract] OR “natural killer cell”[Title/Abstract] OR “natural killer cells”[Title/Abstract] OR “NK cell”[Title/Abstract] OR “NK cells”[Title/Abstract] OR NKG2A[Title/Abstract] OR CD94[Title/Abstract] OR TIGIT[Title/Abstract] OR KIR[Title/Abstract] OR KIR2DL1[Title/Abstract] OR “TIM-3”[Title/Abstract] OR TIM3[Title/Abstract] OR “LAG-3”[Title/Abstract] OR LAG3[Title/Abstract] OR “innate lymphoid cell”[Title/Abstract] OR “innate lymphoid cells”[Title/Abstract] OR ILC1[Title/Abstract] OR “PD-1”[Title/Abstract] OR PD1[Title/Abstract] OR “PD-L1”[Title/Abstract] OR PDL1[Title/Abstract] OR “CTLA-4”[Title/Abstract] OR CTLA4[Title/Abstract] OR CD155[Title/Abstract] OR PVR[Title/Abstract] OR CD112[Title/Abstract] OR NECTIN2[Title/Abstract] OR “HLA-E”[Title/Abstract] OR “Qa-1”[Title/Abstract] OR macrophag*[Title/Abstract] OR neutrophil*[Title/Abstract] OR “myeloid-derived suppressor cell”[Title/Abstract] OR “myeloid-derived suppressor cells”[Title/Abstract] OR MDSC[Title/Abstract] OR MDSCs[Title/Abstract] OR “regulatory T cell”[Title/Abstract] OR “regulatory T cells”[Title/Abstract] OR Treg[Title/Abstract] OR Tregs[Title/Abstract] OR “dendritic cell”[Title/Abstract] OR “dendritic cells”[Title/Abstract] OR fibros*[Title/Abstract] OR “hepatic stellate cell”[Title/Abstract] OR “hepatic stellate cells”[Title/Abstract] OR collagen[Title/Abstract] OR “extracellular matrix”[Title/Abstract] OR “single-cell”[Title/Abstract] OR “single cell”[Title/Abstract] OR “spatial transcriptom*”[Title/Abstract] OR “imaging mass cytometr*”[Title/Abstract] OR “immune microenvironment”[Title/Abstract] OR “immune escape”[Title/Abstract] OR “immune tolerance”[Title/Abstract] OR immunosuppress*[Title/Abstract] OR dysfunction[Title/Abstract] OR exhaustion[Title/Abstract]) AND (“2016/01/01”[Date - Publication]: “2026/05/27”[Date - Publication])

### 2.2 Web of science search string

TS=((“alveolar echinococcosis” OR “hepatic alveolar echinococcosis” OR “Echinococcus multilocularis” OR “E. multilocularis”) AND (“natural killer*” OR “NK cell*” OR NKG2A OR CD94 OR TIGIT OR KIR OR KIR2DL1 OR “TIM-3” OR TIM3 OR “LAG-3” OR LAG3 OR “innate lymphoid cell*” OR ILC1 OR “PD-1” OR PD1 OR “PD-L1” OR PDL1 OR “CTLA-4” OR CTLA4 OR CD155 OR PVR OR CD112 OR NECTIN2 OR “HLA-E” OR “Qa-1” OR macrophag* OR neutrophil* OR “myeloid-derived suppressor cell*” OR MDSC* OR “regulatory T cell*” OR Treg* OR “dendritic cell*” OR fibros* OR “hepatic stellate cell*” OR collagen OR “extracellular matrix” OR “single-cell” OR “single cell” OR “spatial transcriptom*” OR “imaging mass cytometr*” OR “immune microenvironment” OR “immune escape” OR “immune tolerance” OR immunosuppress* OR dysfunction OR exhaustion))

Evidence was synthesized narratively according to thematic and mechanistic relevance. No meta-analysis or pooled quantitative synthesis was performed. Direct NK evidence was distinguished from indirect microenvironmental support wherever possible. Evidence categories were assigned qualitatively using five features: NK-cell specificity, model and compartment, a measured functional endpoint, perturbation or depletion/rescue evidence, and lesion-level relevance. Strong evidence required direct NK functional data plus perturbation or convergent human/animal support; moderate evidence required a direct functional association without complete causal validation; limited evidence reflected phenotypic or model-level clues with weak NK specificity; and very limited evidence denoted indirect tissue-context inference. These categories are qualitative evidence labels rather than meta-analytic scores.

## 3 Hepatic immune microenvironment in AE: from protective inflammation to immune tolerance

The hepatic immune microenvironment in AE is dynamic rather than uniformly suppressive. During early infection, parasite establishment can be opposed by innate immune mechanisms, including macrophage-mediated recognition and elimination, complement-related responses and inflammatory cell recruitment. Recent work suggests that macrophage trogocytosis may eliminate *E. multilocularis* during an early establishment window, supporting the idea that the timing and quality of innate immunity influence whether infection is controlled or becomes chronic (14). This observation supports the view that AE progression represents an immunological transition rather than an immediate state of tolerance.

As AE progresses, the immune landscape becomes increasingly regulatory and spatially organized. Treg expansion is one of the best-supported tolerance mechanisms. Depletion of FoxP3-positive Tregs improves control of larval *E. multilocularis* infection and promotes co-stimulation and Th1/Th17 immunity in experimental models (15). Parasite secreted products can also expand host regulatory T cells, indicating that immunoregulation is not merely a passive consequence of chronic inflammation but can be actively shaped by parasite-derived factors (16). Checkpoint pathways further reinforce this tolerogenic state. PD-1/PD-L1 blockade modifies host responses in murine AE (17, 18), whereas TIGIT, CTLA-4 and LAG-3 have been linked to T-cell exhaustion, immune imbalance and disease progression (19–22). For NK biology, these findings support a suppressive tissue context, but they do not by themselves prove direct Treg-to-NK inhibition in AE. Treg-derived IL-10 and TGF-β remain plausible constraints on NK cytokine production that require AE-specific validation.

Myeloid cells provide another layer of immune remodeling. Macrophages can support early parasite control, but chronic infection is associated with polarization toward suppressive or fibrosis-associated states. Recent studies have described hepatic macrophage polarization as a contributor to immune microenvironment remodeling (23), TIM4-positive macrophages as suppressors of proinflammatory responses during chronic infection (24), and macrophage-derived S100A9 as a driver of hepatic fibrosis through SPARC upregulation in hepatic stellate cells (25). Myeloid-derived suppressor cells also appear to be functionally relevant because targeting these cells promotes antiparasitic T-cell immunity and enhances the efficacy of PD-1 blockade (26). Together, these studies suggest that cytotoxic lymphocyte dysfunction in AE may be reinforced by myeloid-derived suppressive signals rather than by checkpoint receptors alone. Direct macrophage-to-NK suppression remains incompletely established *in vivo*, although limited patient-derived co-culture evidence supports a KIR2DL1-linked interaction (41). Direct MDSC-to-NK suppression in AE remains largely untested and should be prioritized for co-culture and spatial validation.

High-dimensional and spatial approaches now provide tissue-level context for these observations. Single-cell analysis of liver-infiltrating lymphocytes from individuals with chronic *E. multilocularis* infection revealed local immune heterogeneity involving exhausted CD8 T cells, Th17 cells, inducible Tregs and innate lymphoid populations (27). Spatiotemporal transcriptomic profiling further suggested a transcriptomically inferred transition from an immune-active state toward negative immune segregation, involving neutrophils, Spp1-positive macrophages and fibroblasts (28). Imaging mass cytometry has extended this spatial view by mapping immune-cell distributions and interactions within AE lesions (29). In 2026, CCR8 was reported to orchestrate an immunosuppressive liver niche that promotes *E. multilocularis* infection, partly through recruitment of Tregs and functionally impaired T cells (30). Collectively, these findings support the interpretation that NK-cell dysfunction occurs within a spatially organized hepatic immune escape niche rather than as an isolated single-cell phenomenon. However, most spatial and single-cell studies have not yet placed NK cells at the center of their analyses. Future spatial studies should therefore map NK localization relative to parasite vesicles, the fibrotic rim, macrophage-rich zones and Treg/DC niches. The tissue-level framework summarized in **Figure 1** integrates these parasite-proximal, myeloid, regulatory and fibrotic features around the NK-cell compartment.

**Figure 1:**
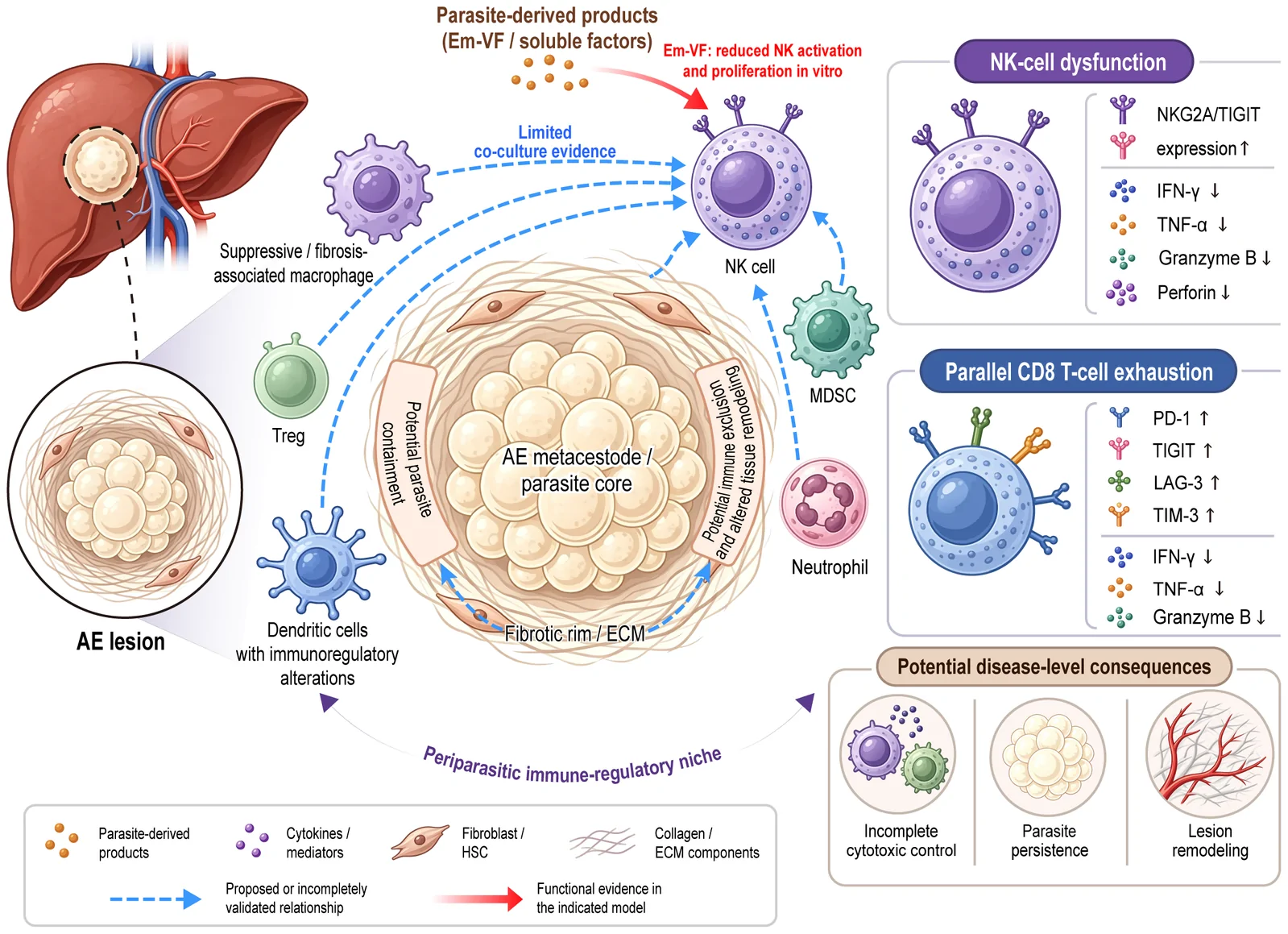
NK-cell dysfunction within the periparasitic immune-regulatory niche in hepatic alveolar echinococcosis. Conceptual organization of parasite-derived products, immune cells and the fibrotic rim surrounding an *Echinococcus multilocularis* metacestode lesion. The solid red arrow denotes experimental evidence that *E. multilocularis* vesicular fluid (Em-VF) reduces NK-cell activation and proliferation *in vitro*. Blue dashed arrows denote proposed or incompletely validated relationships. Proposed links to NK cells involve suppressive/fibrosis-associated macrophages, regulatory T cells (Tregs), dendritic cells with immunoregulatory alterations, myeloid-derived suppressor cells (MDSCs), neutrophils and the fibrotic rim/extracellular matrix (ECM). Macrophage-mediated NK suppression has limited support from *in vitro* co-culture experiments using patient-derived NK cells and EmF-conditioned THP-1 macrophage-like cells; its relevance within AE lesions remains unresolved. The two branches from the fibrotic rim illustrate its potential dual role in parasite containment and immune exclusion with altered tissue remodeling. The right-hand panels summarize reported NK-cell dysfunction and parallel CD8 T-cell exhaustion. Increased receptor expression and reduced effector readouts are drawn from different studies, models and tissue compartments, rather than a single co-occurring phenotype; receptor upregulation alone does not establish pathway activation or exhaustion. Incomplete cytotoxic control, parasite persistence and lesion remodeling are presented as potential disease-level consequences, not a validated linear causal sequence. The model does not imply direct NK-cell killing of metacestode tissue. AE, alveolar echinococcosis; EmF, *E. multilocularis* cyst fluid; HSC, hepatic stellate cell; NK, natural killer; IFN-γ, interferon-γ; TNF-α, tumor necrosis factor-α.

## 4 Liver NK cells and their relevance to parasite control and fibrosis

NK cells are major effector lymphocytes of the liver and bridge innate immune surveillance with adaptive immune regulation (31, 32). In infection and tumor biology, NK cells can rapidly produce IFN-γ and TNF-α, release perforin and granzyme B, express degranulation markers such as CD107a, and engage death-receptor pathways including TRAIL and FasL. These functions can activate macrophages, restrict infected or transformed cells and shape downstream T-cell responses. In the liver, NK cells are also linked to tissue remodeling because they can influence hepatic stellate cell activation and fibrogenesis (33).

The relevance of these functions to AE is conceptually strong but experimentally uneven. The parasite resides primarily in the liver, where NK cells are abundant, and parasite persistence is accompanied by impaired cytotoxic immunity, checkpoint expression and fibrotic lesion remodeling. Current studies indicate defects in both cytokine-related readouts, including IFN-γ and TNF-α, and cytotoxic readouts, including granzyme B, perforin and degranulation-associated markers. These changes should not be interpreted as proof that NK cells directly kill parasite tissue. A more cautious interpretation is that loss of NK-derived cytokines and cytotoxic programs may weaken macrophage activation, cytotoxic lymphocyte cooperation and lesion-level immune pressure. Conversely, NK cells may also participate in lesion containment and fibrosis-related responses, so their role may not be uniformly protective or uniformly pathogenic. This duality is important in AE, where fibrosis can both restrict parasite spread and create a barrier that limits immune-cell penetration and drug access. More broadly, chronic stimulation can produce NK-cell dysfunction, but this terminology should be applied with functional evidence rather than inferred from receptor expression alone (34). In this review, a phenotypic association denotes a change in receptor expression, subset frequency or tissue localization without a linked functional assay; functional impairment denotes a measured reduction in cytokine production, cytotoxicity or degranulation; an exhaustion-like state is reserved for persistent functional impairment accompanied by multiple inhibitory features; and experimentally demonstrated dysfunction requires a direct functional assay plus perturbation, depletion or rescue evidence. Peripheral-blood findings are kept distinct from hepatic-lesion or perilesional tissue findings because these compartments may contain different NK-cell states.

An additional challenge is subset definition. Human liver contains circulating NK cells, CD56dim and CD56bright NK subsets, liver-resident NK-like populations, ILC1-like cells and NKT cells, with overlapping markers and context-dependent functions (31, 32). Mouse studies often use CD49a and DX5 to distinguish liver-resident and conventional NK populations, but these categories do not map perfectly onto human subsets. Studies of NKT cells in *E. multilocularis* infection also highlight the need to avoid conflating NKT-Treg crosstalk with conventional NK-cell biology (35). Accordingly, the term “NK-cell dysfunction” is used when functional impairment is supported, whereas “exhaustion” is not inferred solely from inhibitory-receptor expression. NK–ILC1–NKT boundaries remain an unresolved issue.

### 4.1 Box 1. NK-cell subset ambiguity in hepatic AE

Human conventional NK cells are commonly defined as CD3-negative CD56-positive lymphocytes, but this broad definition contains functionally different populations. CD56dim NK cells are generally more cytotoxic, whereas CD56bright NK cells are more cytokine-producing and immunoregulatory. Liver-resident NK-like populations may express tissue-retention or residency-associated markers such as CD69, CXCR6, CD49a and CD103, although marker combinations vary across studies and species (31). ILC1-like cells overlap with NK cells but differ in developmental and transcriptional programs, including T-bet and Eomes-associated states (32). NKT cells are CD3-positive and should not be merged with conventional NK cells. In hepatic AE, future flow cytometry, imaging and single-cell studies should explicitly separate conventional NK cells, liver-resident NK-like cells, ILC1-like cells and NKT cells before assigning dysfunction to “NK cells” as a whole. Accordingly, CD56-positive or CD161-based measurements alone should be reported as NK/NKT-like or innate-lymphoid evidence unless the panel includes sufficient markers to exclude CD3-positive NKT cells and distinguish conventional NK cells from ILC1-like populations.

## 5 Evidence for NK-cell dysfunction in AE

### 5.1 Early clinical evidence for reduced NK activity

The first clear link between AE and NK cells came from clinical immunology. Vuitton and colleagues reported reduced NK activity in peripheral blood from patients with *E. multilocularis* infection and described immune-cell subpopulations in the periparasitic granuloma (6). Although this study predates modern NK subset analysis and checkpoint biology, it established an important observation: NK activity is altered in human AE. The same work also highlighted the complex cellular organization of the periparasitic lesion, including macrophages, T cells and myofibroblasts, anticipating the later concept of a structured immune-stromal niche.

This early evidence has two implications. First, NK-cell involvement has long been recognized in AE but has not been systematically integrated into the broader immunological framework. Second, reduced NK activity was identified before the mechanisms of NK inhibitory receptors in AE were defined. Modern studies of NKG2A, TIGIT and related pathways can therefore be understood as mechanistic elaborations of a long-standing but incompletely integrated clinical observation.

### 5.2 Parasite-derived products directly impair NK activation

Direct parasite-mediated suppression of NK cells is supported by studies of *E. multilocularis* vesicular fluid. Bellanger and colleagues showed that vesicular fluid inhibits NK-cell activation and proliferation *in vitro*, including reduced CD69 expression and impaired proliferative responses (7). These findings suggest that NK dysfunction may arise, at least in part, from direct parasite-derived immunomodulatory signals before the full complexity of the chronic tissue microenvironment is established.

Subsequent work showed that vesicular fluid can induce immune checkpoint proteins *in vitro*, including PD-1, PD-L1, LAG-3 and TIM-3 (8). This does not establish that each molecule directly suppresses NK cells *in vivo*, but it connects parasite-derived products to a checkpoint-enriched phenotype. Together, these studies support a model in which *E. multilocularis* products may directly impose inhibitory pressure on NK cells and other immune populations, while also priming the broader microenvironment for tolerance.

### 5.3 NKG2A-mediated NK dysfunction

NKG2A is one of the most convincing inhibitory receptors associated with NK-cell dysfunction in AE. In a C57BL/6 mouse model, *E. multilocularis* inoculation reduced hepatic NK-cell frequency and NK-derived IFN-γ, decreased CD49a-positive DX5-negative liver-resident NK cells, and increased NKG2A expression (9). Importantly, NK-cell depletion increased parasite load, suggesting that NK cells contribute to host protection rather than simply reflecting bystander inflammation (9).

Human and translational evidence has further strengthened the case for NKG2A involvement. A 2025 study in hepatic AE patients reported decreased NK-cell function associated with high NKG2A expression, including altered NK subsets and reduced effector molecules in lesion-associated tissue (12). Another 2025 study showed that blocking NKG2A during *E. multilocularis* infection partially relieved impairment of host NK-cell function, increasing functional readouts such as IFN-γ, TNF-α and granzyme B (13). Collectively, these findings support a functional role for NKG2A beyond phenotypic association and identify it as an inhibitory receptor amenable to experimental blockade in AE-associated NK dysfunction, although clinical translation remains unproven. These findings should be interpreted in a compartment-specific manner, because blood- and lesion-associated NK cells may represent distinct functional states.

Thus, NKG2A currently represents the most coherent inhibitory receptor candidate associated with NK-cell dysfunction in AE across mouse, human and blockade-based studies. The current evidence supports an NKG2A-associated NK phenotype, but the lesion-level source, spatial distribution and functional engagement of its ligand axis remain unresolved.

### 5.4 TIGIT/CD155 and cytotoxic NK dysfunction

TIGIT provides the strongest parallel inhibitory pathway to NKG2A. In patients and a mouse model with hepatic *E. multilocularis* infection, TIGIT-positive NK cells increased, whereas cytotoxic and inflammatory molecules including granzyme B, perforin, IFN-γ and TNF-α decreased (10). TIGIT blockade or deficiency improved NK function and reduced metacestode growth in experimental settings (10). This study directly connects inhibitory receptor expression, NK functional impairment and parasite immune escape, supporting TIGIT as a major receptor-side candidate in an NK-oriented framework for AE.

The relevance of TIGIT is reinforced by studies of CD155, one of its key ligands. CD155 has been proposed as a therapeutic target in AE using an *E. multilocularis* infection mouse model (36). In addition, an IL-22-STAT3-CD155 axis has been implicated in AE-related immune exhaustion and therapeutic potential (37). The NK-specific functional evidence comes from the patient and mouse TIGIT study (10). By contrast, the AE-specific CD155 and IL-22-STAT3-CD155 studies primarily provide ligand-side or broader T-cell-centered context (36, 37), and do not by themselves demonstrate TIGIT-mediated dysfunction in NK cells. A cautious interpretation is that TIGIT/CD155 may represent a shared cytotoxic checkpoint context in AE, linking NK dysfunction with parallel T-cell exhaustion, while direct NK-specific ligand engagement remains incompletely resolved.

### 5.5 Other inhibitory receptors: inhibitory KIRs, TIM-3 and LAG-3

Beyond NKG2A and TIGIT, several inhibitory receptors may contribute to NK dysfunction in AE, although the strength of evidence varies. Bayindala and colleagues reported high surface expression of inhibitory killer immunoglobulin-like receptors on NK cells during *E. multilocularis* infection, including KIR2DL1-associated changes in human liver NK cells and Ly49A-associated changes in mice (11). Chinese PubMed-indexed studies have also examined TIM-3 expression in splenic NK cells after *E. multilocularis* infection and the effect of LAG-3 deficiency on NK-cell function and hepatic fibrosis in infected mice (38, 39). These findings expand the inhibitory receptor landscape but should be treated as emerging evidence. A mouse study of TIM-3/Galectin-9 further linked this pathway to stage-dependent Th1/Th2 cytokine remodeling during persistent infection, although the experiments were centered on CD4-positive T cells rather than NK cells (40). A separate patient-derived and THP-1 co-culture study reported that EmF-conditioned macrophage-like cells reduced NK-cell IFN-γ and TNF-α production through a KIR2DL1-linked mechanism, with partial restoration after KIR2DL1 blockade; this supports a macrophage-NK interaction but remains limited by the surrogate macrophage system and *in vitro* design (41).

The strength of evidence differs across these inhibitory pathways. NKG2A and TIGIT are supported by functional evidence and, in the case of NKG2A, blockade data. By contrast, inhibitory KIR/KIR-related pathways, TIM-3, and LAG-3 are supported by more limited or context-dependent evidence and therefore remain candidate rather than established NK-regulatory mechanisms in AE. These findings also suggest that NK-cell dysfunction is unlikely to be governed by a single inhibitory receptor. The receptor-level evidence architecture is summarized in **Figure 2**, which distinguishes receptor-side NK functional evidence from candidate NK-related mechanisms and broader checkpoint context.

**Figure 2:**
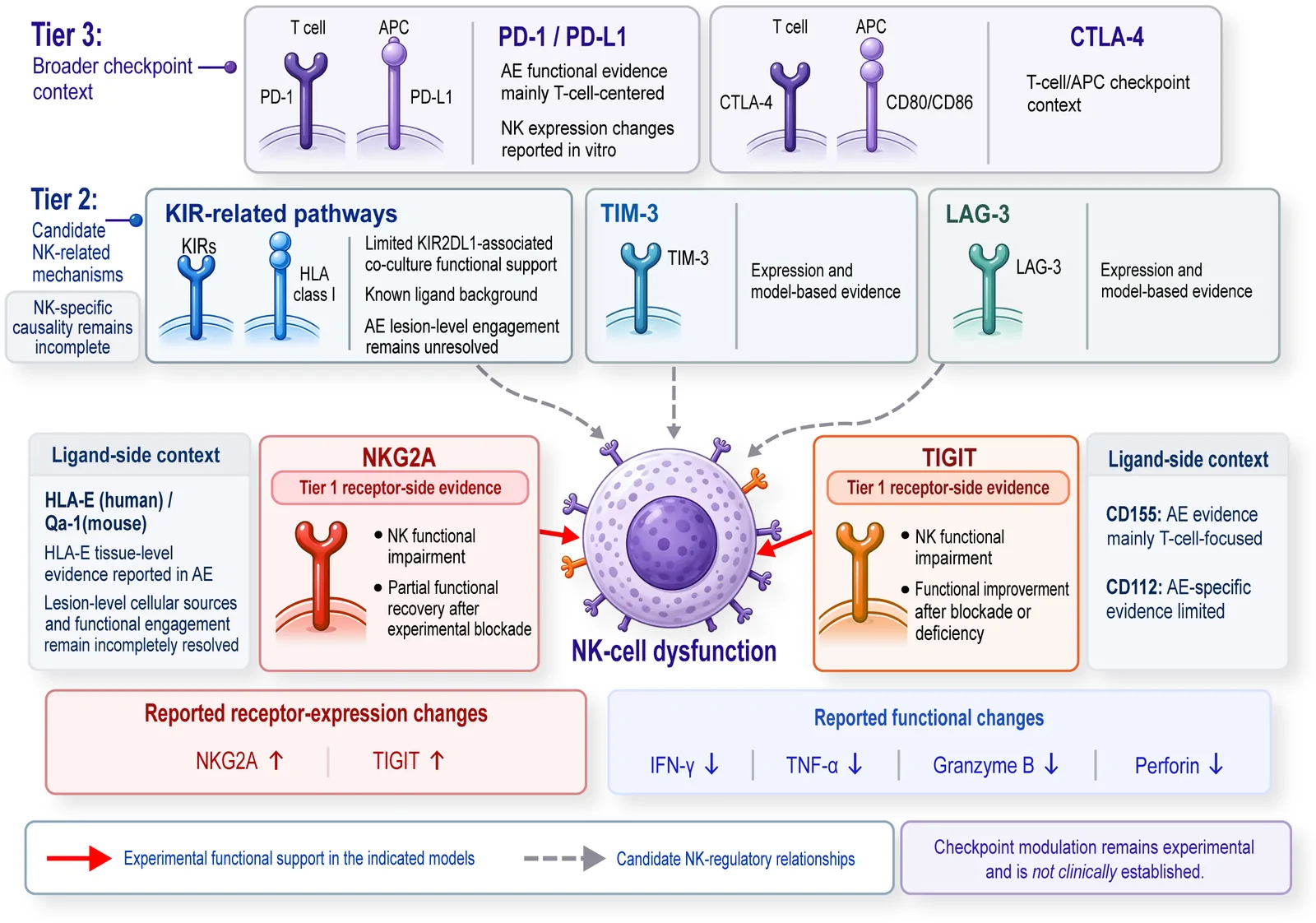
Evidence hierarchy of inhibitory receptors associated with NK-cell dysfunction in alveolar echinococcosis. Checkpoint-related evidence is organized into three qualitative tiers according to the criteria described in Section 2. The underlying studies are summarized in **Table 1** and **Supplementary Table 1**. Tier 1 is restricted to receptor-side functional evidence for NKG2A and TIGIT. This includes NK functional impairment, partial functional recovery after NKG2A blockade, and functional improvement after TIGIT blockade or deficiency. Separate gray panels show ligand-side context. HLA-E tissue-level evidence has been reported in AE, but lesion-level cellular sources, spatial distribution and functional engagement remain incompletely resolved; Qa-1 is the corresponding mouse ligand. AE-specific CD155 studies are predominantly T-cell-focused, whereas evidence for CD112 is limited. Tier 2 comprises KIR-related pathways, TIM-3 and LAG-3. KIR2DL1 has limited co-culture functional support, whereas TIM-3 and LAG-3 have expression and model-based evidence; NK-specific causality remains incomplete. HLA class I is shown as known KIR ligand background, not as a validated pairing in AE lesions. Tier 3 provides broader T-cell/APC checkpoint context for PD-1/PD-L1 and CTLA-4. *In vitro* NK expression changes have been reported for PD-1/PD-L1, whereas CTLA-4 is not depicted as a direct NK inhibitory mechanism. Solid arrows indicate experimental functional support in the indicated models; dashed arrows indicate candidate NK-regulatory relationships. Some blockade, deficiency or depletion experiments are not NK-exclusive. The lower panels distinguish receptor-expression changes from functional changes reported across studies, models and compartments; receptor upregulation alone does not establish pathway activation or exhaustion. Interpretation also depends on study-specific discrimination of NK cells from ILC1 and NKT populations. The tiers are qualitative evidence categories rather than quantitative scores, therapeutic rankings, or measures of clinical efficacy. Checkpoint modulation remains experimental. AE, alveolar echinococcosis; APC, antigen-presenting cell; KIR, killer cell immunoglobulin-like receptor; NK, natural killer; ILC1, group 1 innate lymphoid cell; NKT, natural killer T cell; IFN-γ, interferon-γ; TNF-α, tumor necrosis factor-α.

### 5.6 Receptor-ligand sources: an unresolved mechanistic gap

Inhibitory receptor expression alone does not define a suppressive pathway. Mechanistic characterization of an NK checkpoint axis in AE requires identification of the relevant ligand sources within the lesion. For NKG2A, this means defining the HLA-E pathway in human tissue, or the Qa-1-related axis in mouse models, because the NKG2A-HLA-E pathway is an established inhibitory checkpoint in NK and cytotoxic lymphocyte biology (42). Aierken and colleagues reported increased HLA-E staining in lesion-proximal human liver tissue (13), providing tissue-level ligand-side support. However, this does not identify the contributing hepatocytes, cholangiocytes, macrophages, dendritic cells or stromal cells, or establish their functional engagement with NKG2A-positive NK cells. Thus, the principal unresolved issue is the cell-resolved localization and functional engagement of HLA-E within AE lesions.

For TIGIT, the ligand-side evidence is more developed because CD155 has already been studied as a therapeutic target in AE models (36), and IL-22-STAT3-CD155 signaling has been linked to immune dysfunction (37). The TIGIT-CD155/CD112 axis is also well established as an inhibitory checkpoint in broader immunology (43). The cellular source of CD155 still requires clearer spatial resolution. It could arise from stressed hepatic parenchymal cells, myeloid cells or stromal populations within the fibrotic niche, but this remains to be tested directly. For KIR-related pathways, ligand-side evidence is weaker still, because KIR recognition depends on HLA class I contexts such as HLA-C that have not been systematically mapped in AE lesions. Future work should therefore pair NK receptor phenotyping with ligand localization, ideally using multiplex imaging, spatial transcriptomics or sorted-cell validation. Importantly, the presence of CD155 in an AE model does not by itself establish TIGIT engagement on NK cells; receptor-side NK function and ligand-side tissue biology should therefore be graded separately.

## 6 Cellular networks potentially shaping NK dysfunction in AE

### 6.1 Myeloid cells: strong AE evidence, limited direct NK support

Myeloid remodeling is likely to be a major upstream influence on NK dysfunction, although direct causal links remain incompletely defined. Macrophages in AE can support early parasite control, but chronic infection is associated with macrophage polarization and suppressive or fibrosis-associated states (14, 23–25). TIM4-positive macrophages have been reported to suppress proinflammatory responses and maintain chronic AE infection (24), whereas macrophage-derived S100A9 can promote hepatic fibrosis through SPARC in hepatic stellate cells (25). These macrophage states may create a cytokine and stromal context rich in IL-10, TGF-β, checkpoint ligands and extracellular matrix signals, all of which are plausible constraints on NK activation. A small patient-derived co-culture study provides limited direct functional support for this link: EmF-conditioned THP-1 macrophage-like cells reduced NK-cell IFN-γ and TNF-α production, and KIR2DL1 blockade partially restored these readouts (41). However, the surrogate macrophage system, small cohort and absence of lesion-level ligand mapping prevent this result from establishing an *in vivo* macrophage-to-NK mechanism.

MDSCs add another layer to this suppressive network. Targeting myeloid-derived suppressor cells promotes antiparasitic T-cell immunity and enhances the efficacy of PD-1 blockade (26). Although this work is mainly framed around T cells, it supports the broader concept that myeloid-derived suppression limits cytotoxic immunity in AE. Because NK cells are sensitive to myeloid cytokines, metabolic suppression and checkpoint ligand expression, MDSC-rich environments may indirectly impair NK effector function.

Neutrophils are also prominent in AE lesions and spatial immune models. They may contribute to early defense, tissue injury or lesion segregation depending on disease stage. Spatiotemporal transcriptomic profiling implicates neutrophils together with Spp1-positive macrophages and fibroblasts in a transcriptomically inferred transition from an immune-active state toward negative immune segregation (28). For NK biology, the key implication is not that neutrophils directly suppress NK cells in AE, which remains to be tested, but that chronic neutrophil-rich inflammation and stromal barriers may separate cytotoxic cells from parasite tissue and thereby weaken effective immune attack. In a naturally resistant Wistar rat model, PMN depletion compromised early oncosphere resistance, whereas NK-cell depletion alone did not, underscoring the stage- and model-specific nature of innate-cell contributions (44).

### 6.2 Tregs: strong tolerance evidence, plausible NK suppression

Tregs are among the clearest immunoregulatory populations in AE. Experimental depletion of FoxP3-positive Tregs improves parasite control and enhances co-stimulation and Th1/Th17 immunity (15). FoxP3-positive Tregs have also been proposed as potential immunotherapeutic targets against primary *E. multilocularis* infection (45), and parasite secreted products can expand host Tregs (16). These findings support the concept of parasite-driven tolerance rather than nonspecific immune exhaustion alone.

How Tregs constrain NK cells in AE remains less directly established than their effects on T-cell immunity. However, IL-10, TGF-β, CTLA-4 and PD-1/PD-L1 pathways are known immunoregulatory mechanisms that can limit cytotoxic responses. The 2026 CCR8 study is especially relevant because it explicitly describes a liver immunosuppressive niche that promotes *E. multilocularis* infection through accumulation of Tregs and functionally impaired T cells (30). Current evidence does not establish CCR8/CCL1 as a direct NK-suppressive axis. Rather, it provides tissue-level evidence for a tolerogenic niche in which NK dysfunction may arise or persist.

### 6.3 Dendritic cells: altered in AE, NK priming unproven

Dendritic cells can shape NK function through IL-12, IL-15, IL-18, type I interferons and contact-dependent activation. In AE, hepatic dendritic cells show immunological alterations in patients and mouse models (46), and IDO1 signaling has been implicated in orchestrating immune tolerance in infected mice (47). PD-L1-related pathways and checkpoint blockade studies further indicate that antigen-presenting cells participate in the immunoregulatory network (17, 18).

Current AE studies do not establish a direct DC-to-NK causal pathway. Tolerogenic DC states could nevertheless reduce NK activation by weakening inflammatory cytokine delivery and supporting Treg- and checkpoint-rich environments. DCs therefore remain a component of the proposed NK-involved suppressive context, although their most direct experimental endpoints have involved T cells.

### 6.4 CD8 T cells: parallel cytotoxic exhaustion rather than direct NK regulation

CD8 T-cell dysfunction provides the closest adaptive parallel to NK-cell dysfunction. Inoculum size and chronic antigenic burden influence T-cell tolerance and exhaustion in experimental AE (48). TIGIT blockade can reverse aspects of T-cell exhaustion in murine models (19), and CTLA-4, PD-1 and LAG-3 pathways have been associated with immune suppression or disease progression (20–22, 49). A 2026 study further reported functional reprogramming of hepatic CD8 T cells during AE, including changes in cytotoxic, memory and exhausted states (50). A separate patient study also associated TIM-3-positive CD8+ T cells with reduced perforin, granzyme B and IFN-γ production, with partial *in vitro* recovery after TIM-3 blockade. These findings provide parallel evidence of cytotoxic-lymphocyte dysfunction but are not NK-specific (51).

NK cells and CD8 T cells share effector molecules such as IFN-γ, TNF-α, granzyme B and perforin, and they can be constrained by overlapping checkpoint axes, including TIGIT/CD155 and PD-1/PD-L1. Therefore, NK dysfunction and CD8 T-cell exhaustion should be viewed as parallel defects in cytotoxic immunity rather than as evidence that one compartment directly causes dysfunction in the other. These parallel defects place NK-cell dysfunction within a broader tissue-level impairment of cytotoxic immunity in AE.

## 7 Fibrosis, immune exclusion and NK-cell function

Fibrosis is a defining feature of hepatic AE and has a dual role. On one hand, periparasitic fibrosis can be interpreted as a host containment response that limits parasite spread. On the other hand, fibrotic encapsulation and extracellular matrix deposition can create physical and biochemical barriers that restrict immune-cell access to the lesion core, contributing to immune exclusion and chronic persistence. However, direct extrapolation from tumor immunology may not fully capture the disease-specific role of fibrosis in AE. In AE, the fibrotic rim may serve at least three disease-specific functions: limiting parasite expansion, restricting immune-cell penetration into parasite-proximal regions, and altering drug diffusion or chronic inflammatory maintenance. This dual or even tripartite role is central to the proposed NK-oriented model.

Multiple studies support the importance of fibrosis-associated remodeling in AE. Macrophage-derived S100A9 can drive liver fibrosis through SPARC upregulation in hepatic stellate cells (25). CCL2–CCR2 blockade attenuated hepatic fibrosis and reduced parasite burden in an *E. multilocularis*-infected mouse model, implicating chemokine-myeloid-stromal pathways in lesion progression (52). IL-33/ST2 pathway inhibition combined with albendazole can affect hepatic fibrosis and immune regulation (53). These studies do not directly establish NK-fibrosis causality, but they show that fibrosis is an immunologically active component of the AE niche rather than a passive scar.

NK cells are relevant because they can influence hepatic stellate cell activation and fibrotic remodeling in liver disease more broadly. In AE, NKG2A-associated mouse data reported that NK depletion increased parasite load but reduced periparasitic fibrosis (9). This observation suggests that NK cells may contribute to parasite control while also interacting with fibrotic containment. The relationship is therefore unlikely to be linear. NK dysfunction may weaken cytokine-mediated antiparasitic pressure, whereas residual NK activity or NK-driven inflammatory crosstalk may still influence fibrotic architecture. Future studies should decouple antiparasitic immunity from fibrosis remodeling by measuring parasite burden, NK effector state, stellate-cell activation and collagen architecture in parallel. Thus, fibrosis-associated immune exclusion should not be treated as uniformly detrimental in AE: the same matrix response may constrain parasite expansion while reducing immune-cell access and altering drug distribution. The net effect is expected to depend on lesion location, disease stage and the balance between containment and exclusion.

## 8 An evidence-supported conceptual model of NK-oriented immune escape

The evidence reviewed above supports a conceptual model in which NK-cell dysfunction is embedded in a broader hepatic immune escape niche. This model has three evidence layers. The first layer is direct NK evidence. Reduced NK activity in AE patients, Em-VF-mediated suppression of NK activation, NKG2A-associated functional decline, TIGIT-associated NK dysfunction and partial restoration of NK effector function after checkpoint blockade provide the strongest support for NK involvement (6–13). Collectively, these findings support the inclusion of NK-cell dysfunction within current models of AE immune escape without establishing NK cells as the dominant immune driver of the disease.

The second layer is microenvironmental support. Treg expansion, checkpoint-enriched T-cell exhaustion, MDSC activity, altered dendritic-cell states, suppressive macrophage phenotypes and spatial immune segregation are well supported in AE, but most of these pathways have not yet been shown to directly suppress NK cells *in vivo* (15–30, 46–50). They should therefore be interpreted as components of a plausible NK-involved suppressive context rather than as fully established NK-specific mechanisms. This distinction preserves the separation between established NK-specific mechanisms and tissue-level associations.

The third layer is translational inference. NKG2A and TIGIT currently represent the most compelling receptor-side candidates for NK-directed immunomodulation. HLA-E/Qa-1 and CD155/CD112 provide complementary but still incomplete ligand-side support, whereas inhibitory KIR-related, TIM-3, LAG-3 and PD-1/PD-L1-related mechanisms remain more context-dependent or less NK-specific (10–13, 17–22, 36–39). The model therefore does not imply that all checkpoint pathways are equivalent, nor that checkpoint blockade is clinically ready for AE. Accordingly, the available evidence can be organized into three tiers: Tier 1 receptor-side evidence includes NKG2A and TIGIT, which have AE-relevant NK functional evidence, whereas the corresponding HLA-E/Qa-1 and CD155/CD112 ligand-side evidence remains incomplete; Tier 2 includes inhibitory KIRs/KIR-related pathways, TIM-3 and LAG-3, which have expression or model-based clues but weaker NK-specific causality; Tier 3 includes PD-1/PD-L1 and CTLA-4, which are mainly T-cell or APC-centered in the current AE literature. The condensed study-level evidence map is provided in **Table 1**, and a full study-by-study evidence map, including species/model, sample size, compartment, NK-cell identification, functional endpoints, perturbations and limitations, is available in **Supplementary Table 1**. This hierarchy provides a framework for prioritizing mechanistic experiments: mapping NK subsets in human lesions, testing direct effects of parasite products on NK states, defining macrophage/Treg/DC-to-NK crosstalk, localizing receptor ligands, and separating parasite-control effects from fibrosis-remodeling effects.

**Table 1:** Evidence hierarchy of NK-cell dysfunction and immunosuppressive networks in AE.

Evidence module	Main finding	NK specificity	Mechanistic status	Evidence strength/limitation
Early human NK evidence	PBMC NK activity was reduced	NK-associated PBMC cytotoxicity readout; not purified NK cells	Functional association	Historical study; limited mechanistic resolution
Parasite product-NK interaction	Em-VF inhibited NK activation and proliferation	NK-specific *in vitro* evidence	Direct functional suppression *in vitro*	*In vitro* evidence; tissue context lacking
Checkpoint induction by parasite products	Em-VF induced PD-1, PD-L1, LAG-3 and TIM-3	NK and T-cell phenotyping; no NK functional rescue	Phenotypic association	NK-specific functional causality limited
NKG2A in mouse AE	Hepatic NK frequency and IFN-γ declined; NKG2A increased; NK depletion increased parasite load	NK-focused mouse evidence; depletion not fully NK-exclusive	Functional association with depletion evidence	Mouse model; ligand source unresolved
TIGIT-associated NK dysfunction	TIGIT-positive NK cells increased; cytotoxic mediators declined; TIGIT blockade improved NK function	NK-focused functional evidence; systemic intervention not NK-exclusive	Functional checkpoint evidence	Clinical translation not yet established
Inhibitory KIR	Human KIR2DL1 and murine Ly49A were associated with reduced NK effector readouts	NK phenotypic and functional association	Functional association without receptor perturbation	Ligand source, gating details and receptor-specific causality limited
Human hepatic NKG2A evidence	High NKG2A associated with decreased NK function	NK-specific human tissue evidence	Functional association	Sample size and causality limitations
NKG2A blockade	NKG2A blockade partially restored NK effector function	NK-focused functional evidence; systemic blockade not NK-exclusive	Experimental rescue	Preclinical or early translational evidence
Treg-mediated tolerance	Treg depletion improved control; parasite products expanded Tregs	Microenvironment-only for NK	Indirect support	NK effects mostly inferential
MDSC/checkpoint network	MDSC targeting enhanced antiparasitic T-cell immunity and PD-1 blockade	Mainly T-cell/APC-level evidence	Indirect support	Direct NK link not established
CCR8/CCL1 niche	CCR8 promoted Treg and impaired T-cell accumulation in liver	Microenvironment-only for NK	Niche-level functional evidence	Direct NK link not yet established
Spatial immune landscape	AE lesions show lymphocyte heterogeneity and spatial immune organization	Tissue-context evidence	Spatial or phenotypic association	Not all studies are NK-focused; NK localization needs refinement
Macrophage-NK KIR2DL1 crosstalk	EmF-conditioned THP-1 cells reduced NK-cell IFN-γ/TNF-α; KIR2DL1 blockade partially restored function	NK-specific patient-derived co-culture evidence	Functional association with partial blockade rescue	Small cohort; THP-1 surrogate macrophages; ligand source unresolved
TIM-3/Galectin-9 cytokine remodeling	TIM-3/Galectin-9 increased during persistent AE and accompanied stage-dependent IFN-γ/IL-4 changes	CD4 T-cell-centered, not NK-specific	Mechanistic association	No direct NK assay; liver-injection model; parasite burden not directly tested
Early PMN defense	PMN depletion enabled early oncosphere invasion and hepatic metacestode development in resistant Wistar rats	Early innate evidence, not NK-specific	Depletion-based *in vivo* evidence	Rat oral-infection model; early establishment stage; depletion specificity limits
TIM-3-associated CD8 T-cell dysfunction	TIM-3-positive CD8 T cells showed lower cytotoxic readouts in active AE; blockade partially restored function *in vitro*	CD8 T-cell-centered parallel evidence	Functional association with *in vitro* rescue	Peripheral blood; no direct NK analysis; *in vitro* intervention

This simplified main-text table highlights the evidence hierarchy most relevant to the NK-oriented model. For transparency, **Supplementary Table 1** records species/model, sample or animal number when available, tissue or compartment, NK-cell identification strategy, principal functional endpoint, perturbation or depletion/rescue design and the main limitation for each study. The complete study-by-study evidence map is cited here as **Supplementary Table 1**.

The NK-oriented model should therefore be regarded as a structured hypothesis integrating direct NK findings with established tissue-level immunoregulation rather than as evidence that NK cells are the dominant immune driver of AE. It also identifies specific priorities for future mechanistic investigation: which NK subset is dysfunctional, which inhibitory receptor is functionally dominant, which neighboring cell type provides the relevant ligand or cytokine signal, and whether restoring NK function improves parasite control without worsening hepatic injury.

## 9 Therapeutic implications and unresolved questions

Checkpoint modulation represents a potential experimental implication of AE-associated NK-cell dysfunction, although current evidence remains preclinical. NKG2A blockade partially restores NK-cell function in *E. multilocularis* infection (13), and TIGIT blockade or deficiency improves NK responses and limits metacestode growth in experimental systems (10). CD155-targeted work and the IL-22-STAT3-CD155 axis further support the relevance of TIGIT ligand biology (36, 37). Together, these studies identify NKG2A and TIGIT as the most compelling receptor-side candidates, with emerging ligand-side support for CD155.

Other checkpoint pathways are important but less NK-specific. PD-1/PD-L1 blockade can modify murine AE responses (17, 18), CTLA-4 blockade can reverse aspects of T-cell exhaustion (20), and LAG-3 studies suggest stage- and subset-dependent immunoregulatory effects (21, 22, 39). Current evidence is insufficient to classify these pathways as NK-directed therapeutic targets in AE. They are better considered components of a broader checkpoint network influencing adaptive and innate cytotoxicity.

Therapeutic immunomodulation in AE will require caution. Enhancing cytotoxic immunity could improve parasite control but may also increase hepatic inflammation, tissue damage or fibrosis-associated remodeling. Immune checkpoint modulation in AE remains preclinical and must be evaluated for hepatic immunopathology, fibrosis remodeling and compatibility with albendazole. Albendazole remains central to treatment, and immune-directed strategies would most plausibly be explored as adjunctive approaches in carefully defined experimental or translational settings. At present, the principal translational value of this framework lies in defining experimentally testable pathways and priorities rather than in supporting immediate clinical intervention. In particular, checkpoint modulation should be tested with parallel monitoring of parasite burden, liver injury, inflammatory cytokines, collagen architecture, hepatic stellate-cell activation and albendazole tolerability. A favorable NK functional readout alone would not establish a favorable therapeutic balance.

Several questions remain unresolved. First, which NK subset is most affected in AE: circulating NK cells, liver-resident NK cells, CD56dim NK cells, CD56bright NK cells, ILC1-like cells or NKT-like populations? Second, do NKG2A and TIGIT mark the same dysfunctional NK state or distinct suppressive programs? Third, are parasite-derived products sufficient to induce NK dysfunction, or is sustained myeloid/Treg/DC crosstalk required? Fourth, does NK dysfunction primarily promote parasite persistence, fibrosis remodeling or both? Fifth, can checkpoint modulation be safely combined with antiparasitic treatment without worsening hepatic injury? Addressing these questions will require careful separation of human tissue evidence, mouse model causality, *in vitro* parasite product assays and spatial immune mapping. Longitudinal sampling, multiplex tissue imaging, flow cytometry with NK/ILC-discriminating panels, and ex vivo co-culture systems using parasite products and defined hepatic immune populations would be particularly useful.

## 10 Conclusion

Accumulating evidence indicates that NK-cell dysfunction is an under-integrated component of AE immunology. Available evidence includes reduced NK activity in patients, direct suppression by parasite-derived vesicular fluid, NKG2A- and TIGIT-associated functional impairment, and partial restoration of NK effector function after checkpoint blockade in experimental settings.

This review positions NK-cell dysfunction as an under-recognized cytotoxic node within the established myeloid-, Treg-, T-cell-, and fibrosis-associated immune escape network of hepatic AE. The strongest NK-specific receptor-side evidence currently supports NKG2A and TIGIT, whereas ligand-side evidence for HLA-E/Qa-1 and CD155/CD112 remains incomplete. KIR-related, TIM-3, LAG-3, PD-1/PD-L1, and CTLA-4 pathways should therefore be considered candidate or broader checkpoint mechanisms unless NK-specific causality is demonstrated. Future work should map NK subsets, ligand sources, spatial localization and functional rescue in combination with antiparasitic therapy.

## References

[1] Wen H Vuitton L Tuxun T Li J Vuitton DA Zhang W . Echinococcosis: advances in the 21st century. Clin Microbiol Rev. (2019) 32:e00075-18. doi: 10.1128/CMR.00075-18 30760475 PMC6431127

[2] Vuitton DA Gottstein B . Echinococcus multilocularis and its intermediate host: a model of parasite-host interplay. J BioMed Biotechnol. (2010) 2010:923193. doi: 10.1155/2010/923193 20339517 PMC2842905

[3] Ciomaga BF Hogea MO Muntean AA Muntean MM Popa MI Popa GL . Alveolar echinococcosis in the early 2020s: a systematic review. Pathogens. (2026) 15:132. doi: 10.3390/pathogens15020132 41754385 PMC12943347

[4] Gottstein B Soboslay P Ortona E Wang J Siracusano A Vuitton DA . Immunology of alveolar and cystic echinococcosis (AE and CE). Adv Parasitol. (2017) 96:1–54. doi: 10.1016/bs.apar.2016.09.005 28212788

[5] Wang J Gottstein B . Immunoregulation in larval Echinococcus multilocularis infection. Parasite Immunol. (2016) 38:182–92. doi: 10.1111/pim.12292 26536823

[6] Vuitton DA Bresson-Hadni S Laroche L Kaiserlian D Guerret-Stocker S Bresson JL . Cellular immune response in Echinococcus multilocularis infection in humans. II. Natural killer cell activity and cell subpopulations in the blood and in the periparasitic granuloma of patients with alveolar echinococcosis. Clin Exp Immunol. (1989) 78:67–74. PMC1534601 2805425

[7] Bellanger AP Mougey V Pallandre JR Gbaguidi-Haore H Godet Y Millon L . Echinococcus multilocularis vesicular fluid inhibits activation and proliferation of natural killer cells. Folia Parasitol. (2017) 64:29. doi: 10.14411/fp.2017.029 28906255

[8] Bellanger AP Courquet S Pallandre JR Godet Y Millon L . Echinococcus multilocularis vesicular fluid induces the expression of immune checkpoint proteins *in vitro*. Parasite Immunol. (2020) 42:e12711. doi: 10.1111/pim.12711 32171024

[9] Abulizi A Shao Y Aji T Li Z Zhang C Aini A . Echinococcus multilocularis inoculation induces NK cell functional decrease through high expression of NKG2A in C57BL/6 mice. BMC Infect Dis. (2019) 19:792. doi: 10.1186/s12879-019-4417-1 31500589 PMC6734356

[10] Zhang C Wang H Li J Hou X Li L Wang W . Involvement of TIGIT in natural killer cell exhaustion and immune escape in patients and mouse model with liver Echinococcus multilocularis infection. Hepatology. (2021) 74:3376–93. doi: 10.1002/hep.32035 34192365

[11] Bayindala Huang H Gao S Xu X . Echinococcus multilocularis induces surface high expression of inhibitory killer immunoglobulin-like receptor on natural killer cells. Allergol Immunopathol. (2021) 49:78–86. doi: 10.15586/aei.v49i5.465 34476926

[12] Abulizi A Tuergan T Shalayiadang P Zhang C Zhang R Jiang T . Hepatic alveolar echinococcosis infection induces a decrease in NK cell function through high expression of NKG2A in patients. Front Immunol. (2025) 16:1563248. doi: 10.3389/fimmu.2025.1563248 40421027 PMC12104054

[13] Aierken A Aierken A Aimulajiang K Abulizi A Yuan Z Liu C . Blocking NKG2A in Echinococcus multilocularis infection partially relieves impairment of NK cell function of the host. Cell Mol Life Sci. (2025) 82:297. doi: 10.1007/s00018-025-05838-y 40751812 PMC12317963

[14] Yang N Li L Xue J Zhang X Zhang H Shi B . The critical window for host defense: macrophage trogocytosis eliminates Echinococcus multilocularis at the early establishment stage. Front Immunol. (2026) 17:1734332. doi: 10.3389/fimmu.2026.1734332 41909673 PMC13017266

[15] Wang J Muller S Lin R Siffert M Vuitton DA Wen H . Depletion of FoxP3(+) Tregs improves control of larval Echinococcus multilocularis infection by promoting co-stimulation and Th1/17 immunity. Immun Inflammation Dis. (2017) 5:435–47. doi: 10.1002/iid3.181 28621034 PMC5691311

[16] Nono JK Lutz MB Brehm K . Expansion of host regulatory T cells by secreted products of the tapeworm Echinococcus multilocularis. Front Immunol. (2020) 11:798. doi: 10.3389/fimmu.2020.00798 32457746 PMC7225322

[17] Wang J Jebbawi F Bellanger AP Beldi G Millon L Gottstein B . Immunotherapy of alveolar echinococcosis via PD-1/PD-L1 immune checkpoint blockade in mice. Parasite Immunol. (2018) 40:e12596. doi: 10.1111/pim.12596 30315719 PMC6587932

[18] Jebbawi F Bellanger AP Lunstrom-Stadelmann B Rufener R Dosch M Goepfert C . Innate and adaptive immune responses following PD-L1 blockade in treating chronic murine alveolar echinococcosis. Parasite Immunol. (2021) 43:e12834. doi: 10.1111/pim.12834 33754355

[19] Zhang C Lin R Li Z Yang S Bi X Wang H . Immune exhaustion of T cells in alveolar echinococcosis patients and its reversal by blocking checkpoint receptor TIGIT in a murine model. Hepatology. (2020) 71:1297–315. doi: 10.1002/hep.30896 31410870

[20] Yang Y Wuren T Wu B Cheng S Fan H . The expression of CTLA-4 in hepatic alveolar echinococcosis patients and blocking CTLA-4 to reverse T cell exhaustion in Echinococcus multilocularis-infected mice. Front Immunol. (2024) 15:1358361. doi: 10.3389/fimmu.2024.1358361 38605966 PMC11007148

[21] Li D Ainiwaer A Zheng X Wang M Shi Y Rousu Z . Upregulation of LAG3 modulates the immune imbalance of CD4+ T-cell subsets and exacerbates disease progression in patients with alveolar echinococcosis and a mouse model. PloS Pathog. (2023) 19:e1011396. doi: 10.1371/journal.ppat.1011396 37172058 PMC10208502

[22] Ainiwaer A Li D Liu W Deng B Li Y Xiao W . LAG3 constrains anti-parasitic response by effector CD4(+) T-cell in early Echinococcus multilocularis-infected mice. Parasites Vectors. (2026) 19:122. doi: 10.1186/s13071-026-07246-y 41680821 PMC12998186

[23] Xu H Wang Y Mi L Ren L Wang Z . Polarization of hepatic macrophages in alveolar echinococcosis and its role in remodeling the immune microenvironment. Trop Med Infect Dis. (2026) 11:96. doi: 10.3390/tropicalmed11040096 42042957 PMC13119565

[24] Wang L Liu Y Ma Y Zhou X Aibibula M Zhang X . TIM4+macrophages suppress the proinflammatory response to maintain the chronic alveolar echinococcosis infection. Front Cell Infect Microbiol. (2025) 15:1600686. doi: 10.3389/fcimb.2025.1600686 40606633 PMC12213818

[25] Yang N Shi B Xue J Zhang H Chu J Zhang X . Macrophage-derived S100A9 drives liver fibrosis in Echinococcus multilocularis infection by up-regulating secreted protein acidic and rich in cysteine (SPARC) in hepatic stellate cells. Int J Biol Macromol. (2026) 346:150624. doi: 10.1016/j.ijbiomac.2026.150624 41619879

[26] Zhang C Wang H Aji T Li Z Li Y Ainiwaer A . Targeting myeloid-derived suppressor cells promotes antiparasitic T-cell immunity and enhances the efficacy of PD-1 blockade. Nat Commun. (2024) 15:6345. doi: 10.1038/s41467-024-50754-7 39068159 PMC11283557

[27] Jiang T Sun W Aji T Shao Y Guo C Zhang C . Single-cell heterogeneity of the liver-infiltrating lymphocytes in individuals with chronic Echinococcus multilocularis infection. Infect Immun. (2022) 90:e0017722. doi: 10.1128/iai.00177-22 36317875 PMC9670881

[28] Ou Z Li L Ren P Zhou TT He F Chen J . Spatiotemporal transcriptomic profiling reveals the dynamic immunological landscape of alveolar echinococcosis. Adv Sci. (2025) 12:e2405914. doi: 10.1002/advs.202405914 39985260 PMC12079354

[29] Ma Y Zhang Y Zhang T Zhang L Wang Z Yang Z . Imaging mass cytometry of the immune microenvironment in alveolar echinococcosis. Front Cell Infect Microbiol. (2026) 16:1759455. doi: 10.3389/fcimb.2026.1759455 42180255 PMC13194580

[30] Hou J Fan H . CCR8 orchestrates an immunosuppressive niche in the liver to promote Echinococcus multilocularis infection. PloS NeglTrop Dis. (2026) 20:e0014018. doi: 10.1371/journal.pntd.0014018 41790619 PMC12965546

[31] Peng H Wisse E Tian Z . Liver natural killer cells: subsets and roles in liver immunity. Cell Mol Immunol. (2016) 13:328–36. doi: 10.1038/cmi.2015.96 26639736 PMC4856807

[32] Vivier E Artis D Colonna M Diefenbach A Di Santo JP Eberl G . Innate lymphoid cells: 10 years on. Cell. (2018) 174:1054–66. doi: 10.1016/j.cell.2018.07.017 30142344

[33] Radaeva S Sun R Jaruga B Nguyen VT Tian Z Gao B . Natural killer cells ameliorate liver fibrosis by killing activated stellate cells in NKG2D-dependent and tumor necrosis factor-related apoptosis-inducing ligand-dependent manners. Gastroenterology. (2006) 130:435–52. doi: 10.1053/j.gastro.2005.10.055 16472598

[34] Bi J Tian Z . NK cell exhaustion. Front Immunol. (2017) 8:760. doi: 10.3389/fimmu.2017.00760 28702032 PMC5487399

[35] Yarahmadov T Wang J Sanchez-Taltavull D Rojas CAA Brodie T Buchi I . Primary infection by E. multilocularis induces distinct patterns of cross talk between hepatic natural killer T cells and regulatory T cells in mice. Infect Immun. (2022) 90:e0017422. doi: 10.1128/iai.00174-22 35862712 PMC9387288

[36] Zhang X Li L Sun T Yang N Liu H Chu J . CD155 as a therapeutic target in alveolar echinococcosis: insights from an Echinococcus multilocularis infection mouse model. Front Microbiol. (2025) 16:1624387. doi: 10.3389/fmicb.2025.1624387 40666808 PMC12259647

[37] Li L Zhang X Yang N Liu H Xue J Chu J . IL-22-STAT3-CD155 axis in alveolar echinococcosis: a pivotal role in immune exhaustion and therapeutic potential. Front Immunol. (2026) 16:1674904. doi: 10.3389/fimmu.2025.1674904 41562076 PMC12813123

[38] Shi Y Abidan A Li D Zibigu R Wang M Zheng X . Effect of Echinococcus multilocularis infection on Tim3 expression in spleen natural killer cells of mice. Zhongguo Xue Xi Chong Bing Fang Zhi Za Zhi. (2023) 35:366–73. doi: 10.16250/j.32.1374.2023064 37926471

[39] Zibigu R Abidan A Adilai D Li Y Kang X Yu Q . Effect of LAG3 deficiency on natural killer cell function and hepatic fibrosis in mice infected with Echinococcus multilocularis. Zhongguo Xue Xi Chong Bing Fang Zhi Za Zhi. (2024) 36:59–66. doi: 10.16250/j.32.1374.2024013 38604686

[40] Li S Zhu Y Wang S Li Y Pang N An M . Tim-3/Galectin-9 signaling pathway is involved in the cytokine changes in mice with alveolar echinococcosis. Mol Biol Rep. (2022) 49:7497–506. doi: 10.1007/s11033-022-07554-3 35715604

[41] Bayindala Aji T Ran B Jiang T Tongbayier W Shao Y . Macrophages induce natural killer cell dysfunction via KIR2DL1 during Echinococcus multilocularis infection. Trop J Pharm Res. (2019) 18:1527–32. doi: 10.4314/tjpr.v18i7.24

[42] Andre P Denis C Soulas C Bourbon-Caillet C Lopez J Arnoux T . Anti-NKG2A mAb is a checkpoint inhibitor that promotes anti-tumor immunity by unleashing both T and NK cells. Cell. (2018) 175:1731–43.e13. doi: 10.1016/j.cell.2018.10.014 30503213 PMC6292840

[43] Harjunpaa H Guillerey C . TIGIT as an emerging immune checkpoint. Clin Exp Immunol. (2020) 200:108–19. doi: 10.1111/cei.13407 31828774 PMC7160651

[44] Joekel DE Nur S Monne Rodriguez J Kronenberg PA Kipar A LeibundGut-Landmann S . Agranulocytosis leads to intestinal Echinococcus multilocularis oncosphere invasion and hepatic metacestode development in naturally resistant Wistar rats. Parasitology. (2021) 148:53–62. doi: 10.1017/S0031182020002012 33087186 PMC11010136

[45] Wang J Cardoso R Marreros N Muller N Lundstrom-Stadelmann B Siffert M . Foxp3(+) T regulatory cells as a potential target for immunotherapy against primary infection with Echinococcus multilocularis eggs. Infect Immun. (2018) 86:e00542-18. doi: 10.1128/IAI.00542-18 30037796 PMC6204723

[46] Wang H Li Y Yu Q Wang M Ainiwaer A Tang N . Immunological characteristics of hepatic dendritic cells in patients and mouse model with liver Echinococcus multilocularis infection. Trop Med Infect Dis. (2024) 9:95. doi: 10.3390/tropicalmed9050095 38787028 PMC11125766

[47] Meng R Fu Y Zhang Y Mou Y Liu G Fan H . Indoleamine 2,3-dioxygenase 1 signaling orchestrates immune tolerance in Echinococcus multilocularis-infected mice. Front Immunol. (2022) 13:1032280. doi: 10.3389/fimmu.2022.1032280 36439161 PMC9691980

[48] Zhang C Shao Y Yang S Bi X Li L Wang H . T-cell tolerance and exhaustion in the clearance of Echinococcus multilocularis: role of inoculum size in a quantitative hepatic experimental model. Sci Rep. (2017) 7:11153. doi: 10.1038/s41598-017-11703-1 28894272 PMC5593833

[49] Sun T Yang Y Qiu Y Wang T Yang M Shen S . High PD-1 and CTLA-4 expression correlates with host immune suppression in patients and a mouse model infected with Echinococcus multilocularis. Parasites Vectors. (2024) 17:437. doi: 10.1186/s13071-024-06511-2 39456030 PMC11515268

[50] Tang J Qin X Hou S Huo Y Wu P Qian B . Alveolar echinococcosis drives functional reprogramming of hepatic CD8(+) T cells. Front Cell Infect Microbiol. (2026) 16:1747682. doi: 10.3389/fcimb.2026.1747682 41798755 PMC12960575

[51] Zhao H Li B Pang N Li Z Aibibula M Tian F . High expression of Tim-3 in alveolar echinococcosis mediates depletion of CD8+ T cell function. Ann Clin Lab Sci. (2021) 51:827–36. 34921036

[52] Fan B Ma Y Meng M Chen J Qi X Sun Y . Blockade of the CCL2-CCR2 axis attenuates fibrosis and parasite load in vesicular echinococcosis by inhibiting the PI3K-AKT pathway that regulates angiogenesis and hepatic stellate cell apoptosis. Parasites Vectors. (2026) 19:239. doi: 10.1186/s13071-026-07306-3 42057202 PMC13227867

[53] Cheng SL Ma XM Wu BJ Yang YX Fan HN . Therapeutic effects of IL-33/ST-2 pathway inhibition combined with albendazole on hepatic fibrosis and immune regulation in alveolar echinococcosis: *in vivo* and *in vitro* evidence. Parasites Vectors. (2026) 19:177. doi: 10.1186/s13071-026-07355-8 41933369 PMC13104343

