# Supplementary material for: Natural killer cell dysfunction in hepatic alveolar echinococcosis: an under-recognized cytotoxic node in parasite immune escape
Source: Front Immunol. 2026 Oct 19;17:1976611. doi: 10.3389/fimmu.2026.1976611 (PMC13725774; doi:10.3389/fimmu.2026.1976611)
Supplement: Supplementary file 1 [file DataSheet1.pdf]

Supplementary Table 1. Full evidence map of NK-cell dysfunction and broader immunosuppressive networks in AE

Studies are ordered by first citation in the main text. N denotes subjects or animals unless samples, pooled controls or experimental preparations are explicitly indicated. Missing details are identified as source-reporting limitations, not inferred.

Evidence module	Study/year	Species/model	N	Tissue/compartment	NK-cell identification	Main finding	Functional endpoint	Perturbation/depletion/blockade	Evidence limitation
Early human NK evidence	Vuitton et al., 1989 (6)	Human AE; healthy and non-parasitic biliary-disease controls	14 AE overall; NK assay: 9 AE, 12 healthy controls, 12 biliary controls; liver histology: 12 AE	Peripheral-blood PBMC; periparasitic liver granuloma	Bulk PBMC K562 cytotoxicity assay, not purified NK cells; Leu-7/HNK1 phenotyping in 5 patients only	PBMC NK activity was reduced; granulomas were dominated by macrophages, T cells and stromal cells	4-h chromium-release assay against K562 at multiple effector:target ratios; tissue immunophenotyping	No NK depletion or checkpoint blockade	Historical bulk-cell assay; no modern NK/ILC1 discrimination; reduced cytotoxicity does not establish direct parasite killing
Parasite product-NK interaction	Bellanger et al., 2017 (7)	Healthy-donor PBMC and purified NK-cell cultures	17 healthy donors; at least 10 experiments per reported analysis	Peripheral blood; 3- and 6-day in vitro cultures	EasySep NK enrichment; flow gate CD3-negative CD56-positive	Em-VF inhibited NK activation and proliferation; NK viability and measured NK cytokines did not significantly change	CD69; proliferation-dye dilution; viability; NK-supernatant IFN-γ and TGF-β	Em-VF 1 µg/mL on days 0 and 3; IL-2-supported cultures and stimulation controls	Small in vitro effects; no lesion validation, receptor rescue or demonstrated reduction of NK IFN-γ in this experiment
Checkpoint induction by parasite products	Bellanger et al., 2020 (8)	Healthy-donor PBMC and purified NK-cell cultures	22 healthy donors	Peripheral blood; days 3 and 6 in vitro	Purified NK cells gated CD3-negative CD56-positive; CD3-positive CD4-positive T cells assessed separately	Em-VF induced PD-1, PD-L1, LAG-3 and TIM-3 on NK cells; CTLA-4 induction was principally T-cell related	Surface checkpoint expression; soluble checkpoints and culture cytokines; no NK killing/rescue endpoint	Em-VF 1 µg/mL on days 0 and 3; stimulation controls; no checkpoint blockade	Direct NK phenotyping, but receptor induction alone does not demonstrate NK dysfunction or NK-specific causality
NKG2A in mouse AE	Abulizi et al., 2019 (9)	Female C57BL/6 mice; portal-vein inoculation of 2,000 PSCs	3-6 mice/group depending on assay; depletion/fibrosis experiments: 4/group	Liver at 2, 4, 12 and 24 weeks; blood used to check depletion	CD3-negative NK1.1-positive; CD49a-positive DX5-negative resident-like subset	Hepatic NK frequency and IFN-γ declined; NKG2A increased; NK depletion increased parasite load but reduced periparasitic fibrosis	Intracellular IFN-γ/TNF-α/granzyme B; lesion number/size; collagen and α-SMA staining	Anti-NK1.1 (PK136), 150 µg intraperitoneally three times/week from week 12; no NKG2A-specific blockade	Anti-NK1.1 is not exclusively conventional-NK specific; NKG2A association is distinct from NK-depletion evidence; Methods/legends report differing per-assay N
TIGIT-associated NK dysfunction	Zhang et al., 2021 (10)	Human AE; hepatic PSC-infection mouse model including TIGIT-deficient mice	18 AE liver donors; most paired functional analyses: 10; blood: 10 AE + 10 controls; mice: 4-6/group (TIGIT knockout: 5/group)	Blood; close/distant human liver; mouse liver and spleen	Human CD3-negative CD56-positive, CD56/CD16 subsets; mouse CD3-negative NK1.1-positive, CD49a/CD49b subsets	TIGIT-positive NK cells showed impaired effector responses; blockade/deficiency improved NK function and reduced metacestode growth	IFN-γ/TNF-α; granzyme B/perforin; CD107a in mice; lesion weight/foci and ALT/AST	Anti-TIGIT in patient PBMC cultures; systemic anti-TIGIT during weeks 12-24; TIGIT deficiency; IFN-γ-deficient comparator	Human rescue was in PBMC cultures; systemic blockade/global deficiency also affect non-NK cells; NK-specific ligand engagement not isolated
Inhibitory KIR/Ly49 association	Bayindala et al., 2021 (11)	Human paired liver samples; BALB/c mice with intrahepatic metacestode inoculation	17 AE patients; mouse group sizes not stated in the article Methods or figure legends	Close/distant human liver; mouse liver at 4, 12 and 24 weeks	Human CD56-positive NK population with CD3/CD16 panel; complete exclusion gate not shown; murine NK gate insufficiently specified	Human KIR2DL1-positive and mouse Ly49A-positive NK populations were associated with lower effector readouts and fibrosis	Intracellular IFN-γ/TNF-α, granzyme B and perforin; Sirius red/Masson collagen staining	Infection and receptor-positive/negative comparisons; no receptor blockade or genetic perturbation	Functional association, not receptor-specific causality; murine Ly49A is not KIR2DL1; limited gating and N reporting
Human hepatic NKG2A evidence	Abulizi et al., 2025 (12)	Human hepatic AE; paired close/distant lesion tissue	36 tissue donors for histology; fresh-cell phenotyping/function: 10; PET correlation: 12; cohorts must not be summed	Close liver tissue (~0.5 cm) and distant tissue (≥2 cm)	CD3/CD56/CD16 flow panel; CD56dim/CD56bright subsets (Fig. 5); no definitive ILC1 exclusion	NKG2A enrichment associated with impaired NK effector function, lesion activity and fibrosis-related tissue changes	Intracellular IFN-γ, granzyme B, perforin and TNF-α; NKG2A correlations with PET and collagen/α-SMA	Paired tissue and receptor-positive/negative comparisons; no NKG2A blockade in this study	Observational association; endpoint-specific donor counts; tissue NKG2A staining is not exclusively NK-specific
NKG2A blockade	Aierken et al., 2025 (13)	Human AE; healthy-donor NK cultures; female C57BL/6 mice given 2,000 PSCs via portal vein	Blood: 22 AE + 18 controls; paired liver: 8; main mouse assays: 5/group (p-AKT: 4/group); in vitro donor N not specified	Human blood and close/distant liver; mouse liver/spleen; purified blood NK cultures	Flow-cytometric NK analyses and purified NK cultures; full gate/purity not specified in supplied main article (supplementary gating figures referenced)	NKG2A blockade partially restored NK effector function and reduced lesions; proximal human tissue also showed increased HLA-E	IFN-γ/TNF-α, granzyme B/perforin; lesion burden; HLA-E/NKG2A histology; PI3K/AKT tissue assays	Anti-NKG2A in 24-h parasite-protein/NK cultures and in vivo; NK/CD8 depletion arms from week 4 to 12	Systemic antibody is not NK-exclusive; no cell-resolved HLA-E source mapping; incomplete gating/in vitro N reporting; original Table 1 total is internally inconsistent
Treg-mediated tolerance	Wang et al., 2017 (15)	C57BL/6-background DEREG and wild-type mice; secondary intraperitoneal AE	4-5 mice/group per experiment; figure legend reports pooled totals of 8-10/group	Peritoneal exudate and spleen; metacestode tissue; months 1 and 4	Not an NK study; CD4/CD25/FoxP3 Tregs and CD11b/CD11c APCs	Treg depletion improved parasite control and Th1/Th17 responses	Metacestode wet weight; T-cell cytokines/proliferation; APC co-stimulation	Diphtheria toxin in DEREG mice, 110 ng/injection three times/week; short/long depletion schedules	Direct Treg intervention but indirect relevance to NK; intraperitoneal model; Treg rebound/DT limitations
Parasite-induced Treg expansion	Nono et al., 2020 (16)	C57BL/6 secondary AE; OT-II/OT-II.Rag1-deficient T-cell and BMDC cultures	5 infected mice/time point; controls: 15/time point pooled in threes; key co-culture: 3 T-cell and 3 BMDC preparations	Peritoneal exudate; spleen/lymph-node T cells; in vitro cultures	Not an NK study; CD4-positive CD25-positive FoxP3-positive Tregs	Parasite E/S products promoted TGF-β-dependent Treg conversion and IL-10 release; EmACT required host TGF-β for conversion	Treg frequency/conversion; suppressive activity; IL-10 secretion	Parasite E/S or recombinant EmACT; anti-TGF-β (1D11); ALK4/5/7 inhibitor SB431542	No NK functional assay; pooled controls and different experimental units; cannot establish direct Treg-to-NK inhibition
MDSC/checkpoint network	Zhang et al., 2024 (26)	Human AE; WT and PD-1-deficient mouse hepatic infection models	64 liver donors; blood: 33 AE + 34 controls; paired MDSC assay: 11; combination-treatment mice: 5-6/group	Human blood and close/distant liver; mouse liver/spleen; T-cell/MDSC co-culture	No NK-directed analysis; human myeloid panels and mouse CD11b/Gr-1/Ly6C subsets; T-cell functional gates	MDSC targeting enhanced antiparasitic T-cell immunity and efficacy of PD-1 blockade	T-cell proliferation/cytokines; metacestode weight/foci; IDO1-related suppression	Anti-Gr-1 alone or with anti-PD-1; PD-1 deficiency; MDSC/T-cell co-culture and IDO1 inhibition	T-cell/APC-centered; anti-Gr-1 also affects other myeloid populations; NK suppression not directly tested
Human single-cell landscape	Jiang et al., 2022 (27)	Human advanced hepatic AE; scRNA-seq and TCR profiling	4 patients; 11 analyzed samples (Results)	Blood; perilesion and adjacent-normal liver	CD45-positive enrichment; transcriptomic NK/ILC/NKT annotation, not a dedicated functional NK gate	AE lesions showed compartment-specific lymphocyte heterogeneity, including NK/ILC and exhausted T-cell populations	Cell frequencies, transcriptional states and TCR clonality; no direct NK functional endpoint	Observational profiling; no NK blockade/depletion	Small advanced-disease cohort; RNA-based identity does not establish NK dysfunction or cell-contact causality
Spatiotemporal immune landscape	Ou et al., 2025 (28)	BALB/c hepatic PSC infection; Stereo-seq, bulk/scRNA-seq; external human transcriptomes	14 spatial samples; 55 bulk samples; scRNA-seq: 4 tissues from 2 mice; external human cohort: 6 patients	Mouse liver across early infection to 15 months; distal/perilesion tissue	Transcriptomic cell annotation/deconvolution; not a dedicated NK phenotyping or functional study	Spatial neutrophil, Spp1-positive macrophage and fibroblast organization supported an inferred transition toward immune segregation	Gene-expression states, spatial distributions and inferred communication; no direct NK killing assay	Infection time-course and computational comparisons; no NK-targeted perturbation	Spatial samples/cells are not independent animal counts; killing and communication inferred from RNA; indirect NK context

Evidence module	Study/year	Species/model	N	Tissue/compartment	NK-cell identification	Main finding	Functional endpoint	Perturbation/depletion/blockade	Evidence limitation
Human spatial immune landscape	Ma et al., 2026 (29)	Human hepatic AE; imaging mass cytometry with histological validation	21 patients overall; IMC: 6 (3 adults + 3 children); validation: 11 adults + 10 children	Paired close/distant liver tissue and lesion-proximal validation regions	Multiplex metal-tagged antibody panel and cluster annotation; full NK lineage exclusion cannot be inferred from a single marker	AE lesions showed spatially heterogeneous immune/checkpoint patterns; NK/T-cell-associated TIGIT signals supplied tissue context	Cell densities, checkpoint intensity and spatial proximity; neutrophil histology; no direct NK function	Observational tissue profiling; no NK blockade/depletion	Small IMC cohort; regions/cells are not independent patients; proximity is not a demonstrated suppressive interaction
CCR8/CCL1 niche	Hou and Fan, 2026 (30)	Female C57BL/6J WT and CCR8-knockout mice; portal-vein injection of 4,000 PSCs	5 mice/group for reported comparisons	Liver and serum at 2, 12 and 24 weeks	Not an NK study; CD45/CD3/CD4/CD8/CD25/CCR8 and FoxP3 T-cell/Treg panel	CCR8 supported Treg and impaired T-cell accumulation; deficiency reduced lesions and improved T-cell responses	Ultrasound lesion volume; liver weight; ALT/AST; T-cell cytokines and cytotoxic mediators	Whole-animal CCR8 knockout versus WT infection	No human validation or direct NK assay; whole-animal deletion does not isolate Treg-specific causality
TIM-3-associated NK phenotypic changes	Shi et al., 2023 (38)	Female C57BL/6 mice; portal-vein inoculation of 50 or 2,000 E. multilocularis PSCs; saline controls	39 mice: low dose 13 (7/6), high dose 15 (9/6), saline 11 (5/6) at 12/24 weeks	Spleen at 12 and 24 weeks	CD3-negative NK1.1-positive by flow cytometry; no conventional-NK/ILC1 discrimination	Dose/time-dependent TIM-3 expression: increased at 12 weeks with low-dose infection and reduced at 24 weeks with high-dose infection versus controls	TIM-3-positive NK frequency/count and co-expression with 2B4/LAG-3; no direct NK functional assay	Infection-dose/time comparisons; no TIM-3 blockade, knockout or NK depletion	Splenic phenotyping only; expression does not establish receptor activation, functional impairment or exhaustion; no hepatic validation
LAG-3 deficiency and compartment-specific NK responses	Zibigu et al., 2024 (39)	Female C57BL/6 WT and global LAG3-knockout mice; portal-vein inoculation of 3,000 PSCs	12 mice: 6 WT + 6 LAG3-knockout	Liver and spleen at 12 weeks	CD3-negative NK1.1-positive by flow cytometry; no conventional-NK/ILC1 discrimination	LAG3 deficiency increased splenic NK IFN-γ and perilesional collagen; hepatic NK cytokines were not significantly altered	Intracellular IFN-γ/TNF-α/IL-4/IL-10/IL-17A; NK frequency, activation markers and Ki67; Sirius red collagen area	Global LAG3 knockout versus infected WT; no NK-specific deletion, depletion or antibody blockade	Small whole-animal knockout study; compartment-dependent findings; no proof of NK-mediated fibrosis or generalized hepatic NK functional recovery
TIM-3/Galectin-9 cytokine remodeling	Li et al., 2022 (40)	Female BALB/c mice; intrahepatic injection of 1,000 PSCs; CD4 T-cell cultures	112 mice: 56 infected + 56 controls; 7 time-point subgroups per arm	Liver and serum, days 2-270; purified hepatic CD4 T cells in vitro	Not NK-specific; magnetic CD4 T-cell enrichment and TIM-3/CD4 flow analysis	TIM-3/Galectin-9 changes accompanied stage-dependent Th1/Th2 cytokine remodeling	TIM-3/Galectin-9 RNA; IFN-γ and IL-4 in serum/culture; CD4 T-cell phenotypes	Em-VF/Galectin-9 stimulation and anti-TIM-3 in CD4 T-cell cultures	No direct NK analysis; cytokine regulation does not establish parasite clearance or NK receptor causality
Macrophage-NK KIR2DL1 crosstalk	Bayindala et al., 2019 (41)	Patient-derived NK cells; EmF-conditioned THP-1 macrophage-like co-culture	15 AE + 10 healthy controls; figure-level analysis n = 10-15; separate co-culture donor N not explicit	Peripheral blood; 24-h NK/THP-1 co-culture	NK isolation kit; CD3/CD14 exclusion with CD56 assessment; purity >95% reported, but CD56 positivity symbol omitted in Methods	EmF-conditioned THP-1 cells reduced NK IFN-γ/TNF-α; KIR2DL1 blockade partially restored function	NK cytokine secretion; receptor/effector RNA; macrophage-like cell cytokines	EmF conditioning; anti-KIR2DL1 10 µg/mL versus isotype; PMA stimulation	Small cohort; THP-1 surrogate; imperfect marker reporting and unclear co-culture N; ligand source and in vivo interaction unresolved
Early PMN defense	Joekel et al., 2021 (44)	Naturally resistant Wistar rats; oral 1,000 eggs; susceptible BALB/cJRj mouse controls	65 rats in main study: 7 treatment arms ×7 + 4 placebo arms ×4; 4 mouse controls; early histology: 14 additional rats	Intestine at 16-17 h; liver at 10 weeks; blood for depletion validation	Anti-CD161 depletion and blood CD161 flow analysis; not a complete conventional-NK/ILC/NKT exclusion panel	PMN-containing depletion arms permitted metacestode establishment variably; NK depletion alone did not break resistance (0/7)	Intestinal invasion histology; liver metacestodes; blood depletion efficiency	Anti-PMN serum, clodronate liposomes and anti-CD161 alone/in combinations, with placebo controls	Stage/species-specific; CD161 depletion not NK-exclusive; 10/21 is a subset of PMN-containing arms, not total study N; one PMN/macrophage arm had no lesions
TIM-3-associated CD8 T-cell dysfunction	Zhao et al., 2021 (51)	Human AE with peripheral CD8 T-cell assays	62 AE: 33 metabolically active + 29 inactive; 30 healthy controls	Peripheral blood/PBMC and in vitro CD8 T-cell cultures	CD3-positive CD8-positive T cells; no NK-specific gate or assay	TIM-3-positive CD8 T cells showed lower cytotoxic readouts in active AE; blockade partially restored responses in vitro	Perforin, granzyme B and IFN-γ; TIM-3 expression	Galectin-9 stimulation and anti-TIM-3 in vitro	Parallel T-cell evidence, not NK causality; no lesion NK mapping or clinical intervention
